# Recent Advances, Systemic Therapy, and Molecular Targets in Adenoid Cystic Carcinoma of the Head and Neck

**DOI:** 10.3390/jcm12041463

**Published:** 2023-02-12

**Authors:** Ina Dewenter, Sven Otto, Tamara Katharina Kakoschke, Wenko Smolka, Katharina Theresa Obermeier

**Affiliations:** Department of Oral and Maxillofacial Surgery and Facial Plastic Surgery, Ludwig Maximilians University, D-80337 Munich, Germany

**Keywords:** adenoid cystic carcinoma, head and neck tumor, recurrence and prognosis, molecular treatment targets

## Abstract

With an incidence of 3–4.5 cases per million, adenoid cystic carcinoma (ACC) of the head and neck is one of the most common tumors of the parotid and sublingual salivary glands. In the clinical course, ACC is shown to have an aggressive long-term behavior, which leads to the fact that radical surgical resection of the tumor with tumor-free margins remains the “gold standard” in treating ACC. Particle radiation therapy and systemic molecular biological approaches offer new treatment options. However, risk factors for the formation and prognosis of ACC have not yet been clearly identified. The aim of the present review was to investigate long-term experience of diagnosis and treatment as well as risk and prognostic factors for occurrence and outcome of ACC.

## 1. Introduction

Adenoid cystic carcinoma (ACC) of the head and neck is a rare neoplasm frequently arising in salivary glands and related tissue [1], accounting for 1% of all head and neck malignancies [2]. An incidence of 3–4.5 cases per million makes ACC one of the most common malignant tumors of the parotid and sublingual salivary glands [3] as well as the most commonly reported tumor of the minor salivary glands [4]. ACC has also been observed in other regions of the head and neck including the trachea, larynx [5], nasal and paranasal sinus, and lacrimal glands [5,6,7]. Slow local growth and perineural invasion, sporadic regional metastasis, and progressive distant metastasis are classical characteristics of ACC [8]. Correlations between clinical outcome and histological grade have been described [9], but risk factors for the formation and prognosis of ACC have still not yet been clearly identified. As ACC is frequently described as “wolf in sheep’s clothing” due to its slow but uncompromisingly growth and dissemination [10], the identification of molecular biological and clinical-pathological predictors of recurrence and survival are needed for a maximization of treatment outcome.

## 2. Epidemiology

The age at which ACC of the head and neck is diagnosed ranges from 11 to 99 years, with the mean age at diagnosis between 50 and 60 years [11,12,13]. A marginally higher prevalence among women has been observed [14], with a male/female ratio of 0.85:1 [15]. In large population-based studies, ACC accounts for 28% of the total incidence of malignant salivary gland tumors [4], with ACC found in one out of every eight malignant parotid gland tumors [16] and accounting for 40% of submandibular gland carcinomas [17].

## 3. Clinical Appearance

ACC of the head and neck exhibits an aggressive long-term behavior. In early stages, ACC is associated with slow growth and an indolent course. Pain occurs in later onsets due to perineural invasion [18]. Depending on the localization of the tumor, symptoms may vary from facial nerve paralysis (parotid gland) to tumor masses showing ulceration or oroantral fistulas (palate) and nasal obstruction (nasal and paranasal sinus) [19,20]. Staging at the time of diagnosis varies. Around one-third of patients are diagnosed with T4 tumor size initially. In addition, 15 percent of patients show lymph nodal invasion or distant metastases at the time of diagnosis. This results in more than 40 percent of patients with a TNM stage 4 tumor at the time of primary diagnosis. Locoregional and distant recurrence is reported in 30 percent of patients. The latest clinical studies reported a mean time period of 3.4 years between the initial diagnosis of ACC and recurrence. In cases of death due to ACC, the mean time between diagnosis and the registration of death was 5.6 years with a range of 0.4–14.1 years [15]. In summary, ACC is clinically characterized by aggressive local growth, high recurrence rates, and decreased overall survival. Age, BMI, and N stage are described as the three main clinical prognostic factors identified determining event free survival in a prospective multicenter study of 470 patients with ACC [14].

## 4. Diagnostic Imaging

Standard preoperative diagnostic imaging includes magnetic resonance imaging (MRI) or computed tomography (CT). Bone invasion imaging is regularly done by CT diagnostics, whereas soft tissue extension and perineural invasion are assessed in MRI [21,22]. The latest studies have shown that conventional MRI of the head and neck should be complemented by whole-body PET/CT to detect local tumor recurrence, lymph node metastases, or distant metastases [23]. Even more PET/CT imaging using ^68^Ga-labelled FAP inhibitors (^68^Ga-FAPI-PET/CT) detected additional metastases, thereby increasing the accuracy of staging and radiotherapy planning volumes relative to conventional CT/MRI [24]. In addition, 99mTc-MIBI SPECT/CT and FDG PET/CT showed promising results in the detection of distant metastases in ACC of the head and neck [25].

## 5. Histopathology

Three histological patterns (tubular, cibriform, solid) have been described in ACC: The tubular pattern is characterized by simple tubules composed of inner ductal and outer myoepithelial cells. The cibriform pattern predominantly consists of myoepithelial cells with myxoid or hyalinized globules along with isolated solitary ductal elements. Solid nests composed of sheets of basaloid cells are typical for the solid pattern [26]. A combination of the cribriform and tubular pattern is often observed in classical ACC. Solid patterns have been associated with a more advanced tumor stage and worse prognosis [27]. Two histological grading systems have been proposed, reflecting the clinical outcome due to the percentage of the solid component in ACC. Perzin [27] and Szanto [28] defined a staging system including three different stages: low grade (stage 1) without a solid component, intermediate grade (stage 2) with a 1–29% solid component, and high grade (stage 3) with a more than 30% solid component. A more recent staging system proposed by Van Weert [29] and Zhang [30] differentiated only two stages: grade one with no solid component and grade two with a solid component. Cervical nodal metastases as well as distant metastases in ACC patients can be histopathologically detected [31].

Another histological property associated with patient outcomes in ACC is perineural invasion (PNI). PNI is significantly correlated with both distant metastasis and unfavorable disease outcomes [32]. However, due to its microscopic appearance, 3D imaging often fails to detect PNI in patients with ACC [33].

## 6. Therapy and Prognosis

Radical surgical resection of the tumor with tumor-free margins remains the “gold standard” in treating ACC of the head and neck. The possibility of achieving tumor-free margins is limited by factors such as tumor localization (proximity to skull base), histopathologic pattern, and previous treatment. The role of surgical margins was examined by Amit et al. who analyzed 507 cases of head and neck ACC in an international multi-center study. Positive margins were associated with the worst outcome, whereas negative and close margins were associated with improved outcome, regardless of the distance from the tumor. Except the oral cavity, the presence of close margin status resulted in similar outcomes as negative margins for ACC of all sites examined. Therefore, negative margins should be achieved whenever possible [34]. Due to the abovementioned limitations, a combination of surgery with radiation therapy has led to superior results in many studies [35], with a disease-specific survival of 92 percent after five years, and 88 percent after ten years. Disease-free survival (DFS) amounted to 67 percent after 5 years and 61 percent after 10 years. Patients with unresectable ACC or positive surgical margins showed a worse disease-specific survival of 78 percent after five years and 32 percent after ten years, as well as worse disease-free survival (35 percent after 5 years, 8 percent after 10 years) [15]. Chemotherapy as a treatment procedure in ACC has also been reported. Common monotherapy strategies have been implemented with 5-FU [36], cisplatin [37], gemcitabine [38], mitoxantrone [39], epirubicin [40], vinorelbine [41], and paclitaxel [42]. In these studies, objective response ranged from 0 to 70% with a median of 16% [43]. Combination chemotherapy such as the combination of cisplatin, doxorubicin, and cyclophosphamide (CAP) has also been examined in several studies [44,45]. The combination of CAP with 5-fluoruracil showed the longest duration of response in patients [46], whereas the combination of cyclophosphamide, vincristine, and 5-FU [47] showed a rather long duration of response. Still, combined chemotherapy led to a relatively poor outcome compared to the treatment side effects (nausea, vomiting, myelosuppression, and neuropathy). An optimum treatment for ACC has not yet been established, probably because of the neurotropic and infiltrative characteristics of the tumor. Disease treatment should be adapted to the initial diagnosis, taking tumor localization, pathological staging, and grading into account [48].

## 7. Particle Radiation Therapy

Heavy-particle radiotherapy using protons, neutrons, or carbon ions has recently emerged as a promising treatment option. Proton treatment has been performed in several studies, using 60 to 76.4 Gray equivalent (GyE) [49,50], giving even patients with unresectable ACC of the head and neck a promising treatment perspective [51]. Case studies observing survival and outcome of inoperable ACC of the head and neck using proton radiation therapy report overall survival (OS) rates of 80% and a three-year progression-free survival of 63% [52]. Furthermore, patients receiving postoperative intensity-modulated proton therapy to a median of 60 GyE in combination with platinum-based chemotherapy showed no evidence of disease at a median of 24.9 months [50]. Recent studies concluded that there is no significant survival advantage in operated patients relative to patients with unresectable ACC when treated with pencil beam scanning proton therapy (PBS PT) [53].

Effects of carbon ion radiotherapy for head-and-neck cancer, especially ACC, have been evaluated in phase II clinical trials, proving the therapeutic effectiveness without severe morbidity of the normal tissues [54,55,56]. More phase II clinical trials are in progress [57]. Results of a combination treatment with intensity-modulated radiotherapy and active raster-scanning carbon ion boost for adenoid cystic carcinoma of the minor salivary glands of the nasopharynx resulted in adequate local control and OS rates with moderate toxicity [58]. Carbon ion radiotherapy with simultaneous integrated boost for head and neck ACC improved the tumor dose conformation while reducing the unintended dose to the low-risk volume and related probability of complications [59]. Even though fast neutron therapy (FNT) seemed to be a promising treatment procedure, it received less consideration in clinical trials due to limited availability, high costs, and challenges with healthy tissue toxicity in treatment cohorts. Side effects such as posttreatment trismus, acute mucositis, and acute xerostomia have been reported [60,61]. Still, the latest case studies including neutron radiation treatment in ACC report a 10-year OS of 62% and osteoradionecrosis rates comparable to that of photon radiation treatment (2–7%) [62]. Recently, early outcomes for a hybrid neutron–proton approach were found to be promising, describing an OS of 93.1% and a PFS of 79.3%. Still, further clinical trials with longer follow-ups and larger patient cohorts are needed for validation [63]. Table 1 shows clinical trials using particle radiation therapy.

## 8. Molecular Biomarkers as a Treatment Target

Molecular markers as a prognostic and therapeutical approach have been of great interest in the last years. Prognostic biomolecular factors can help to identify patients that would benefit from more intense treatment and point out new therapeutic targets. In the following, different gene expression patterns are discussed.

### 8.1. Oncogenes

#### SOX4, C-KIT, VEGF, BDNF, MYB, Wnt/b-Catenin

Cell cycle and apoptosis regulators together with other oncogenes are discussed as having an impact in the pathogenesis of ACC. It was observed that the Sry-related high mobility group (*HMG*) box 4 (*SOX4*) is one of the most highly overexpressed genes in ACC [67]. SOX4 is an essential developmental transcription factor that regulates stemness, differentiation, progenitor development, and multiple developmental pathways [68,69]. Another protein involved in malignant transformation in ACC is the transmembrane tyrosine kinase receptor c-kit. The activation of c-kit promotes cell growth and differentiation [70], and its expression has been shown to correlate with ACC tumor grade [71]. However, clinical trials investigating the effects of c-kit inhibitors in patients have not yet been promising [72].

Furthermore the association of VEGF expression with advanced stage and worse disease-specific survival has been reported [73], along with enhanced microvessel density in ACC, which makes VEGF an important prognosticator of survival and outcome [74,75]. Younes et al. have shown that concomitant inhibition of epidermal growth factor and vascular endothelial growth factor receptor tyrosine kinases reduces the growth and metastasis of human salivary ACC in a mouse model [76], highlighting VEGF and EGF as important therapeutic targets.

As perineural invasion is a frequent complication in ACC, brain-derived neurotrophic factor (BDNF) and nerve growth factor (NGF) have been suspected to facilitate perineural invasion. BDNF has been reported to be uniformly expressed by ACC [77]. Elevated expression of BDNF was found in salivary ACC specimens, which was significantly correlated with the invasion and metastasis [78]. Overexpression of NGF and TrkA in human salivary ACC tissues has also been reported and may constitute a reason for perineural invasion [79]. Furthermore, overexpression of NGF in combination with solid subtype, advanced stage, perineural invasion, recurrence, and extended resection alone have been reported to have worse survival rates showing the relevance of NGF as a prognostic marker [80].

The proto-oncogene *MYB* is associated with apoptosis, cell cycle control, cell adhesion, growth, and differentiation [81]. *MYB* gene fusion or *MYB* protein overexpression occurs in the majority of ACCs, suggesting that MYB might be a potential therapeutic target, but early results have not been promising [82]. Moreover, no significant prognostic differences have been observed between MYB-positive and MYB-negative ACCs, which indicates that MYB is not a good prognostic marker for ACC [83].

Signaling pathways are another target for therapeutic approaches in ACC. Mutations in components of the canonical Wnt/β-catenin signaling pathway have been found in various neoplasms [84,85]. Mutations in components of the Wnt signaling pathway in ACC of the head and neck have already been described [86,87]. In addition reduced membranous expression of β-catenin can be associated with ACC metastasis [88]. The aberrant Wnt/β-catenin signaling pathway enables cancer stem cell renewal, cell proliferation, and differentiation, thus enforcing tumorigenesis and therefore officiating as a therapeutical target in ACC and other head and neck tumors.

### 8.2. Tumorsuppressorgenes

#### p53, p16

Cell cycle regulatory proteins as p53 and p16 and their genes are among the most commonly mutated genes identified in human neoplasms [89]. The p53 protein plays a central role in the expression of genes involved in the regulation of apoptosis and DNA repair. In case damaged DNA is detected in the cell, p53 is phosphorylated, which prevents it from being broken down. The accumulation of p53 induces cell cycle interruption and, if damaged DNA is not repaired properly, apoptosis [90]. Hence, mutations in p53 that limit the protein function ultimately cause replication of damaged DNA, which may result in neoplasms. Studies have shown that p53 mutations in ACC correlate with metastasis and recurrence [91].

The protein p16CDNK2a (p16) is encoded by the gene *CDKN2A* [92,93] and is produced in response to cellular stress signals preventing entry into the S-phase by inhibiting cyclin-dependent kinases (Cdks), which are necessary for the phosphorylation of the retinoblastoma protein (Rb) [94]. Immunohistochemical studies have shown that p16 expression was reduced in ACC cases of higher histological grade of malignancy [95] due to hypermethylation and homozygous deletion as the main inactivation mechanisms of *p16* gene [96]. Therefore, p16 could be used as a prognostic marker for worse survival in ACC of the head and neck and should thus be considered in individual therapy strategies.

### 8.3. Mitochondrial Alterations

The occurrence of mitochondrial alterations and oxidative stress is an important hallmark of tumorigenesis and the development of cancers, including head and neck carcinoma. Mithani et al. showed that 17 out of 22 ACCs carried mitochondrial mutations, most of them occurring in the NADH complex [97]. Along with this finding, it was observed that superoxide dismutase 2 (SOD2) was deregulated in patients with salivary ACC. Up-regulation of SOD2 was associated with distant metastasis and reduced OS and disease-free survival [98]. Reactive oxygen species in high concentrations can be responsible for cell damage, mutations, and tumors [99,100]. For example, hydroxyl anions damage sections of DNA by reacting with purine and pyrimidine bases and with the deoxyribose framework [101], or they are initiators of lipid peroxidation, as a result of which membrane fluidity is reduced, membrane permeability is increased, membrane proteins are damaged, and receptors or ion channels are inactivated or dysregulated [102]. Even more ROS have a significant impact on physiological cell processes such as cellular signal transduction, transcription, and induction of apoptosis [103] and thus affect tumor genesis. Still, further research is needed to determine the effects of the altered antioxidant defense system (as SOD) in ACC.

### 8.4. Immune Microenvironment and Evasion Mechanisms

Expression analysis of immune checkpoints (PD-L1, PD-L2, PD-1, and CTLA-4), immune inhibitory molecule HLA-G, and markers of tumor-infiltrating lymphocytes (TIL) and dendritic cells (DC) in ACC showed low CD8+, GrB+ TIL, CD1a, and CD83 populations, as well as scarce positivity for CTLA-4 and PD-1. In contrast, PD-L2 and HLA-G expression was increased, which suggests that the ACC microenvironment exhibits low immunogenicity, represented by low TIL and DC density. Moreover, an activation of the immune inhibitory proteins/PD-L2 and HLA-G may favor tumor escape from the immune system and partially explain the poor prognosis of ACC [10].

Tumor markers and prognostic markers are expressed differently in each individual, showing that each patient should be considered and treated individually according to personalized medicine.

## 9. Gene Panel Examination and Further Potential Therapeutic Targets

Potential targets identified in adenoid cystic carcinoma point out new directions for further research: the Examinations of the Gene Expression Omnibus (GEO) database was used to explore abnormal coexpression of genes in ACC compared with their expression in normal tissue. The analysis showed that ITGA9 (integrin alpha9) and LAMB1 (laminin subunit beta 1) are important factors regulating the PI3K-Akt pathway. In addition, BAMBI (BMP and Activin membrane-bound inhibitor), a suppressor of TGFβ, serves as an important factor that is involved in the TGF-β signaling pathway and thus acts as a potential target for molecular therapeutic approaches. Further targets described for future studies are SLC22A3 (solute carrier family 22 member 3), FOXP2 (Forkhead box P2), Cdc42EP3 (CDC42 effector protein 3), COL27A1 (collagen type XXVII alpha 1 chain), DUSP1 (dual specificity phosphatase 1), HSPB8 (heat shock protein family B (Small) member 8), ST3Gal4 (ST3 beta-galactoside alpha-2,3-sialyltransferase 4), SPARC (secreted protein acidic and cysteine rich), COL4A2 (collagen type IV alpha 2 chain), PRELP (proline- and arginine-rich end leucine-rich repeat protein), hsa-miR-29-3p, hsa-miR-132-3p, and hsa-miR-708-5p due to regulation of tumorigenesis in ACC [104].

Even more DNA-based next-generation sequencing identified Notch-activating mutations as a promising target in ACC [105]. Notch is involved in both pro- and anti-tumoral effects in the different populations composing the tumor and takes an important role in regulating the crosstalk between the different compartments of the tumor microenvironment [106]. A precise understanding of the contribution of Notch signaling in the different compartments of the TME in ACC is needed in order to design future therapeutic approaches targeting Notch signaling.

## 10. Systemic Molecular Biological Approaches

Within the last 10 years, outcomes of systemic molecular therapy in advanced ACC have been reported in less than 300 patients. Due to the relatively low prevalence of ACC in the head and neck region, most of the clinical trials are small and are only conducted by a single institution. Initial studies using multi-kinase inhibitors such as sunitinib showed no significant observed responses, but the majority of patients showed stable disease with moderate toxic effects [107]. Targeting mTOR resulted in no complete or partial response with a median progression-free survival (PFS) of 11.2 months, with observed tumor shrinkage described in 44% of treated patients [108]. The use of sorafenib (400 mg) showed modest activity in ACC with a 12-month PFS of 46.2%. Sorafenib was associated with significant toxicity, and considering the limited effectiveness, could not be recommended for further evaluation [109]. Therapy using the Akt signaling inhibitor nelfinavir as monotherapy also did not result in a meaningful improvement in clinical outcome [110]. Treating recurrent/metastatic ACC with regorafenib, a tyrosine kinase inhibitor, resulted in stable disease of 6 months in 17 out of 38 patients [111]. In contrast, therapy with dovitinib (another tyrosine kinase inhibitor) showed promising results: tumor shrinkage was observed in 22 out of 32 metastatic or unresectable ACC patients, with one patient with confirmed partial response. Moreover, metabolic activity of the tumor was reduced in 13 patients after dovitinib treatment [112]. In addition, treatment with the multi-kinase inhibitor lenvatinib resulted in partial response in 5 out of 33 enrolled patients and 24 patients (75%) with stable disease, showing promising results while using molecular target therapy [113]. The results are shown in Table 2.

Especially in the latest trials, stable disease was a common result. Still, not all observed patients showed progressive disease initially when included in the study, leading to difficulties in assessment if periods of stable disease are attributable to drug activity or to a non-active period of the disease itself. In all listed studies, median survival was low, which is related to the initial staging (unresectable, distant metastases). Moreover, all listed trials were addressed to advanced and metastatic ACC (none of the included studies addressed to resectable ACC). Therefore, it is not possible to evaluate whether therapeutic response is detectable in earlier stages of ACC. The results are consistent with earlier analyses, which up until now it remains an open question if ACC benefits from treatment with systemic therapy as the clinical outcome by best supportive care (radical surgery with postoperative radiotherapy) may be the same as that by any intervention targeting molecular mechanisms [115]. Still, in patients with unresectable ACC or distant metastases, a participation in clinical trials might be a suitable option that needs to be taken into account individually. As gene panel examination and DNA-based next-generation sequencing offer opportunities to identify future targets, further prospective clinical trials with larger patient cohorts are needed to evaluate more therapeutic potentials.

## 11. Conclusions

There are recommended therapy regimens for ACC of the head and neck, but as shown in the above data, those recommendations show limited efficacy. The activation of immune inhibitory proteins such as PD-L2 and HLA-G may favor tumor escape from the immune system and partially explain the poor prognosis of ACC. Various markers such as SOX4, VEGF, BDNF, and targets of the Wnt/β-catenin signaling pathway and the Notch pathway are expressed in ACC, but not equally in all tumor patients. For this reason, expression profiles of patients should be examined individually, and target therapies should be considered according to a personalized medicine approach. The preferred treatment strategy for the majority of patients with ACC of the head and neck remains as radical surgery with postoperative radiotherapy. Particle radiation therapy, especially proton and carbon ion radiation, were found to improve OS and PFS significantly, thus being a promising future treatment procedure. Systemic molecular biological therapeutic approaches as well as the identification of important prognosis factors offer new treatment options, still requiring further clinical observations.

## Figures and Tables

**Table 1:** Particle radiation therapy. # means number.

Therapy	Authors	# of Patients with ACC	Local Control	OS	Disease-Free Survival	Progression-Free Survival
Protons	Linton et al. [49]	26	2 y LC = 86% (recurrent disease) 95% (primary disease)	57% (recurrent disease), 93% (primary disease)	-	-
Protons + photons	Pommier et al. [54]	23	5 y LC = 93%	77%	56%	-
Protons vs. CIRT	Takagi et al. [64]	34	75%	63%	-	39%
CIRT	Mizoe [55]	69	5 y LC = 73%	68%	-	-
CIRT	Koto [56]	18	5 y LC = 92%	72%	44%	-
CIRT	Akbaba [58]	59	2 y LC = 83%	87%	-	-
CIRT	Mastella [59]	10	-	-	-	-
CIRT	Sulaiman [65]	289	2 y = 88%	94%	-	68%
Neutrons	Douglas [66]	151	5 y LC = 57%	72%	-	-

**Table 2:** Clinical phase II trials investigating systemic molecular biological therapeutic approaches between 2012 and 2022. # means number.

Phase II Trial	Target	Authors	Year	# of Patients with ACC	Objective Response	Progression-Free Survival	Median Survival	Study Design	NOS Score
Sunitinib 37.5 mg	Multikinase inhibitor	Chau et al. [107]	2012	14	0	7.2 months	18.7 months	Clinical trial	4
Everolismus	mTOR	Kim et al. [108]	2014	34	0	11.2 months	-	Clinical trial	4
Sorafenib 400 mg	Multikinase inhibitor	Thomson et al. [109]	2015	23	0	11.3 months	19.6 months	Clinical trial	5
Nelfinavir 1250 mg	Inhibitor of Akt signalling	Hoover et al. [110]	2015	15	0	5.5 months	-	Clinical trial	3.5
Dovitinib 500 mg	FGRF, VEGFR	Keam et al. [112]	2015	32	1	6.0 months	-	Clinical trial	4
Regorafenib 120 mg	FGRF, VEGFR	Ho et al. [111]	2017	38	0	-	-	Clinical trial	3
Lenvatinib	FGRF, VEGFR	Tchekmedyian [113]	2019	32	5	17.5 months	-	Clinical trial	4
Lenvatinib	Multikinase inhibitor	Locati et al. [114]	2020	28	3	9.1 months	27 months	Clinical trial	6

## Data Availability

Not applicable.
